# Dynamic Information Encoding With Dynamic Synapses in Neural Adaptation

**DOI:** 10.3389/fncom.2018.00016

**Published:** 2018-03-27

**Authors:** Luozheng Li, Yuanyuan Mi, Wenhao Zhang, Da-Hui Wang, Si Wu

**Affiliations:** ^1^State Key Laboratory of Cognitive Neuroscience and Learning, Beijing Normal University, Beijing, China; ^2^Center for Brain Sciences, Institute of Military Cognitive and Brain Sciences, Academy of Military Medical Sciences, Beijing, China; ^3^Computer Science Department, Center for the Neural Basis of Cognition, Carnegie Mellon University, Pittsburgh, PA, United States; ^4^School of System Science, Beijing Normal University, Beijing, China

**Keywords:** adaptation, dynamical coding, dynamical synapse, short-term plasticity, balanced input

## Abstract

Adaptation refers to the general phenomenon that the neural system dynamically adjusts its response property according to the statistics of external inputs. In response to an invariant stimulation, neuronal firing rates first increase dramatically and then decrease gradually to a low level close to the background activity. This prompts a question: during the adaptation, how does the neural system encode the repeated stimulation with attenuated firing rates? It has been suggested that the neural system may employ a dynamical encoding strategy during the adaptation, the information of stimulus is mainly encoded by the strong independent spiking of neurons at the early stage of the adaptation; while the weak but synchronized activity of neurons encodes the stimulus information at the later stage of the adaptation. The previous study demonstrated that short-term facilitation (STF) of electrical synapses, which increases the synchronization between neurons, can provide a mechanism to realize dynamical encoding. In the present study, we further explore whether short-term plasticity (STP) of chemical synapses, an interaction form more common than electrical synapse in the cortex, can support dynamical encoding. We build a large-size network with chemical synapses between neurons. Notably, facilitation of chemical synapses only enhances pair-wise correlations between neurons mildly, but its effect on increasing synchronization of the network can be significant, and hence it can serve as a mechanism to convey the stimulus information. To read-out the stimulus information, we consider that a downstream neuron receives balanced excitatory and inhibitory inputs from the network, so that the downstream neuron only responds to synchronized firings of the network. Therefore, the response of the downstream neuron indicates the presence of the repeated stimulation. Overall, our study demonstrates that STP of chemical synapse can serve as a mechanism to realize dynamical neural encoding. We believe that our study shed lights on the mechanism underlying the efficient neural information processing via adaptation.

## 1. Introduction

Adaptation is a general phenomenon that happens when the neural system receives an invariant stimulation and decreases its response. During adaptation, the neural system can dynamically adjust its response property according to the statistics of external inputs (Kohn, 2007; Wark et al., 2007). Previous studies suggest that adaptation underlies how the neural system process information efficiently using its computational resource (such as spikes) (Gutnisky and Dragoi, 2008). During adaptation, the firing rates of neurons first increase dramatically at the onset of the stimulation and then decrease gradually to a low level that is close to the background activity of neurons. Since the repeated stimulation conveys little knowledge, it would seem that it is not necessary for the neural system to encode repeated information. However, our daily experiences indicate this is not true: we can still sense the stimulus in many scenarios, even when the neuronal responses have attenuated. For example, we can view a static image or hear a lasting pure-tone long after our sensory system has adapted (deCharms and Merzenich, 1996). Thus, it prompts a question: during adaptation, how can the neural system sense the existing stimulus with attenuated firing rates?

**Figure 1:**
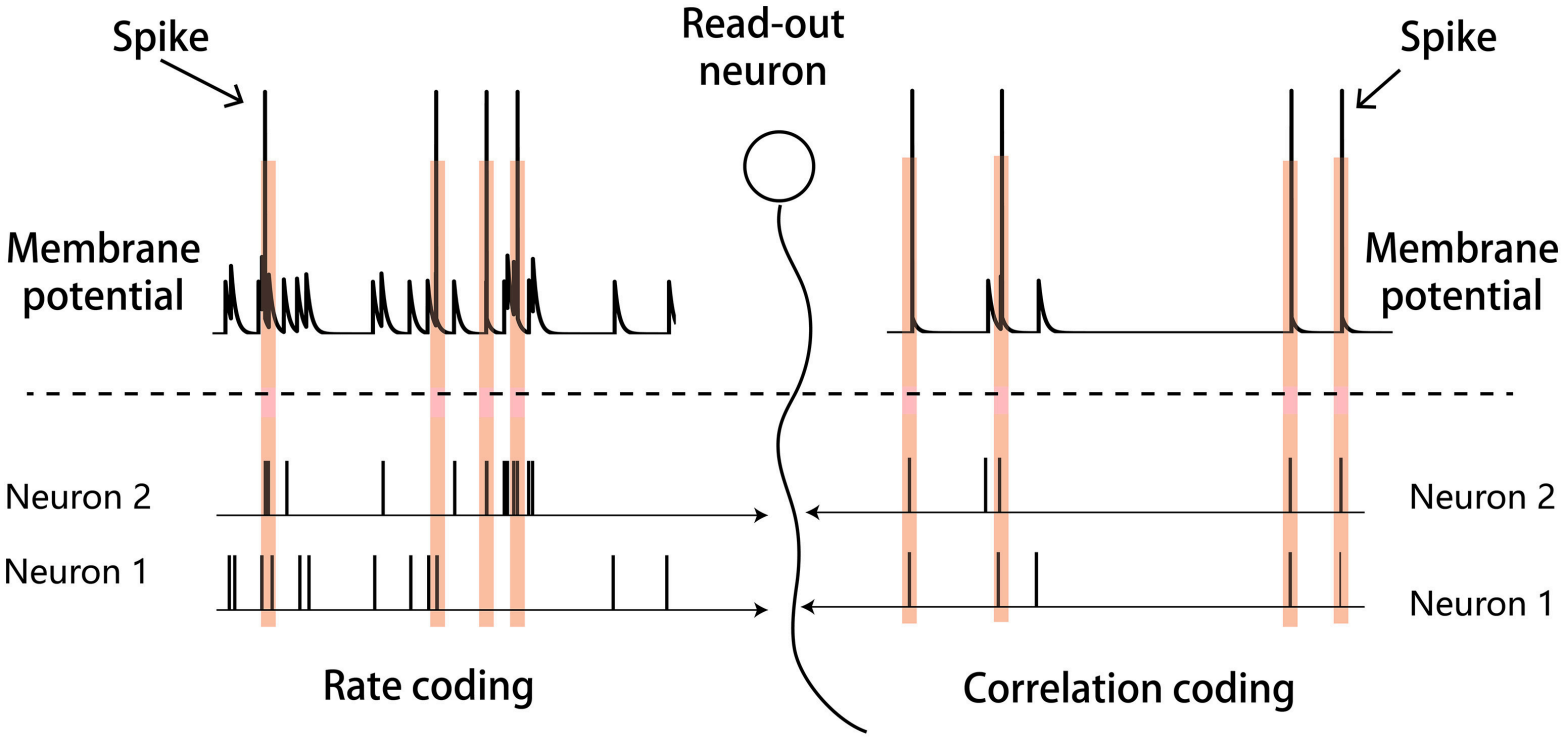
Illustration of the rate and correlation codes. **(Left panel)** Rate coding. Strong and independent firings of individual neurons are sufficient to elicit the downstream read-out neuron. **(Right panel)** Correlation coding. Weak but synchronized firings of neurons are also sufficient to elicit the read-out neuron.

There may exist different strategies for the neural system to encode sensory inputs, and two candidate strategies are rate coding and correlation coding. As illustrated in Figure 1, in rate coding, individual neurons fire strongly and independently to convey the stimulus information to the downstream neuron; whereas, in correlation coding, a group of neurons fire weakly but in a synchronized manner to convey the stimulus information. Both strategies encode the stimulus information, but correlation coding is economically more efficient (consuming less spikes). In the previous study, Xiao et al. proposed that the generic phenomenon of firing rate attenuation in neural adaptation may underlie a dynamical information encoding strategy, i.e., a shift from rate to correlation codes over time (Xiao et al., 2013). Their study was based on the data of bullfrogs' retina neurons (dim detectors) in response to static stimuli. By quantifying the amount of stimulus information encoded in either neuronal firing rates or neuronal pair-wise correlations, they observed that: at the early stage of the adaptation, the stimulus information was mainly encoded in the neuronal firing rates; whereas at the late stage of the adaptation, the stimulus information was mainly encoded in the neuronal correlations. They built a computational model to elucidate the underlying mechanism and suggested that short-term facilitation (STF) of electrical synapses (gap-junctions) is the substrate of dynamical encoding, that is, STF increases neuronal connections in a stimulus-specific manner during the adaptation, which consequently increase the correlations between neurons in spite of their firing rates attenuating (Xiao et al., 2013, 2014). We believe that the idea of dynamical encoding is generally applicable in different forms of neural adaptation in the brain. The previous modeling study only considered electrical synapses between retinal ganglion cells. However, the more common connections between neurons in the sensory cortex are chemical synapses (Connors and Long, 2004). To validate the generality of dynamical encoding, it is necessary to extend the previous work to the case that neurons are connected by chemical synapses.

In this study, we explore the potential role of chemical synapses in implementing dynamical encoding in the neural system. We consider a large-size network, in which neurons are connected by chemical synapses and their efficacy are subject to short-term plasticity (STP). STP can be decomposed into two components: short-term facilitation (STF) and short-term depression (STD), and their relative contributions are varied in different brain regions (Markram et al., 1998; Mongillo et al., 2008), which, in mathematical modeling, can be controlled by choosing different parameters. Here, since we consider information processing in the sensory cortex, we set STP to have a large STD time constant for STD and a small STF utilization increment, consistent with the experimental data (Thomson et al., 2002; Wang et al., 2006). A big difference between electrical and chemical synapses is that: the former is analogical to an constant resister between neurons, whose facilitation can increase the neuronal correlation dramatically; whereas, the latter mediates neuronal interaction via spiking and a single spike only modifies the membrane potential of the post-synaptic neuron mildly, therefore facilitation of a chemical synapse only increases the neuronal correlation slightly. However, when a large-size neural network is considered, weak changes on individual synapses can have a significant impact on the synchronization of the network (Schneidman et al., 2006), which can serve as a substrate to convey the stimulus information. Overall, our study demonstrates that STP of chemical synapses can serve as a substrate to realize dynamical neural encoding.

## 2. Materials and methods

**Figure 2:**
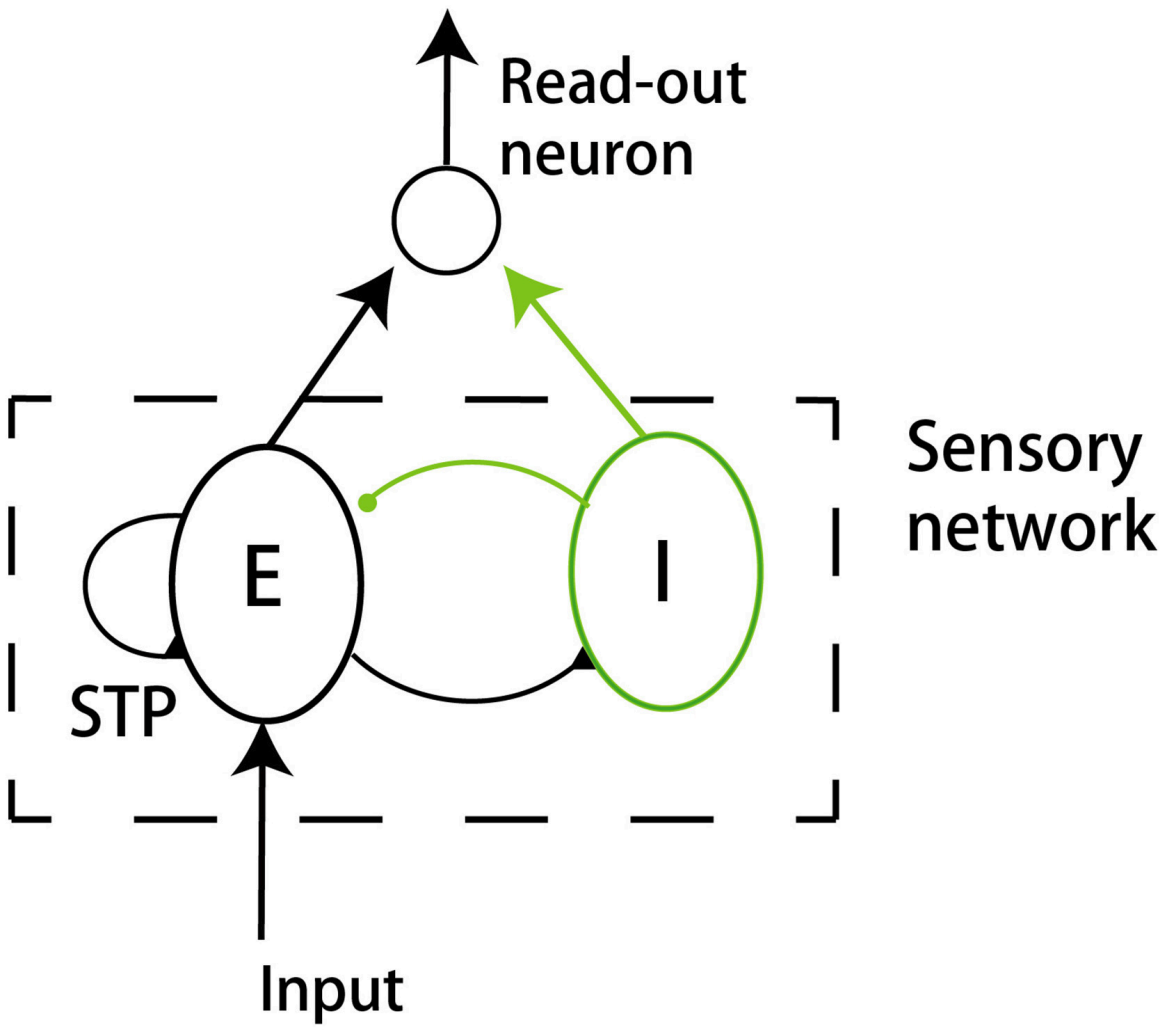
The model structure. The model consists of a sensory network and a downstream read-out neuron. The sensory network is composed of an excitatory neuron group (E) and an inhibitory neuron group (I). The excitatory neurons receive the external input. The synapses between excitatory neurons in the sensory network are subject to STP.

We build up a model to illustrate our ideas of dynamical neural encoding. The model is composed of two parts (Figure 2): a sensory network which simulates the adaptive responses of sensory neurons and STP of neuronal synapses, and a downstream neuron which reads out information of stimulus. An excitatory neuron group(E) and an inhibitory neuron group (I) compose the sensory network. The details of the model are introduced below.

### 2.1. The dynamics of single neurons

For simplicity, we consider that adaptation is caused by the internal dynamics of single neurons. Other mechanisms may also lead to adaptation, such as STD in the feedforward synapses to a neuron (Abbott et al., 1997; Chung et al., 2002), or the interaction between excitation and inhibition neurons (Middleton et al., 2012), but they do not change our main results and hence are not included in the present study. According to the experimental data, a number of cellular mechanisms contribute to the attenuation of neural responses, and they are roughly divided into two classes (Benda and Herz, 2003; Brette and Gerstner, 2005): the spike-triggered mechanism, e.g., the calcium-activated potassium current, and the subthreshold voltage-dependent mechanism, e.g., the voltage-gated potassium current. Without loss of generality, we model these two kinds of adaptation in the dynamics of excitatory neurons as follows: The dynamics of an excitatory neuron is given by,

(1) $$\begin{array}{r} \tau_{m}\frac{dV_{i}}{dt}=-\left(V_{i}-E_{L}\right)+R_{m}\left[I_{syn,i}\left(t\right)-I_{adp,i}\left(t\right)\right], \end{array}$$

where *V*_*i*_ is the membrane potential of neuron *i*, τ_*m*_ the membrane time constant, *E*_*L*_ the leaky reverse potential, *R*_*m*_ the membrane resistance, *I*_*syn, i*_ the synaptic currents received by the neuron, and *I*_*adp, i*_ the adaptive currents generated by the internal mechanism of the neuron. A spike of neuron *i* is generated when *V*_*i*_ reaches a threshold *V*_*th*_, and membrane potential is reset to *E*_*L*_ afterwards.

The synaptic current *I*_*syn, i*_(*t*) consists of external and recurrent parts. The recurrent part *I*_*rec, i*_(*t*) denotes currents from other neurons in the sensory network. The external part *I*_*ext, i*_(*t*) denotes the external stimulus described as a continuous current with a Gaussian white noise.

They are given by,

(2) $$\begin{array}{r} I_{syn,i}\left(t\right)=I_{ext,i}\left(t\right)+I_{rec,i}\left(t\right), \end{array}$$

(3) $$\begin{array}{r} I_{ext,i}\left(t\right)=\mu_{ext}+\sigma_{ext}\eta_{i}\left(t\right), \end{array}$$

where η_*i*_(*t*) satisfies 〈η_*i*_(*t*)〉 = 0, $\langle \eta_{i}\left(t\right)\eta_{j}\left(t^{\prime }\right)\rangle =\delta_{ij}\delta \left(t-t^{\prime }\right)$, and μ_*ext*_ and $\sigma_{ext}^{2}$ are the mean and the variance of the external input, respectively. *I*_*rec, i*_ is the sum of postsynaptic currents from all other neurons in the network received by neuron *i*. The dynamic of *I*_*rec, i*_ is described below.

The adaptation current *I*_*adp, i*_ is given by,

(4) $$\begin{array}{r} \tau_{a}\frac{d}{dt}I_{adp,i}=-I_{adp,i}+A_{p}\left(V_{i}-E_{L}\right)+A_{s}\underset{k}{\sum }\delta \left(t-t_{i}^{k}\right), \end{array}$$

where τ_*a*_ is the time constant of adaptation and $t_{i}^{k}$ the moment of neuron *i* emitting the *k*th spike. The parameter *A*_*p*_ describes the strength of the adaptation under subthreshold voltage and the parameter *A*_*s*_ controls the strength of adaptation due to the spike.

For simplicity, we do not include adaptation currents in inhibitory neurons. The dynamics of an inhibitory neuron is described by Equation (1) except that *I*_*adp, i*_ = 0.

### 2.2. The dynamics of synapses

We only consider STP at synapses between excitatory neurons and other synapses are set as unchanged. Denote *u*_*i*_ to be the release probability of neurotransmitters at each synapse of the pre-synaptic neuron *i*, and *x*_*i*_ the fraction of neurotransmitters available at each synapse. Their dynamics are given by,

(5) $$\begin{array}{r} \tau_{f}\frac{du_{i}\left(t\right)}{dt}=-u_{i}\left(t\right)+U\left[1-u_{i}\left(t\right)\right]\underset{k}{\sum }\delta \left(t-t_{i}^{k}\right), \end{array}$$

(6) $$\begin{array}{r} \tau_{d}\frac{dx_{i}\left(t\right)}{dt}=1-x_{i}\left(t\right)-u_{i}\left(t\right)x_{i}\left(t\right)\underset{k}{\sum }\delta \left(t-t_{i}^{k}\right), \end{array}$$

where τ_*f*_ is the time constant of STF, *U* controls the increment of *u*_*i*_ upon neural firing, and τ_*d*_ is the time constant of STD.

For an excitatory neuron, it receives recurrent inputs from other excitatory and inhibitory neurons, which are written as,

(7) $$\begin{array}{r} \begin{array}{c} \tau_{s}\frac{dI_{rec,i}^{E}\left(t\right)}{dt}=-I_{rec,i}^{E}\left(t\right)+J_{EE}\underset{j\in E}{\sum }w_{ji}u_{j}\left(t\right)x_{j}\left(t\right)\underset{k}{\sum }\delta \left(t-t_{j}^{k}\right)\\ +J_{IE}\underset{j\in I}{\sum }w_{ji}\underset{k}{\sum }\delta \left(t-t_{j}^{k}\right), \end{array} \end{array}$$

where *J*_*EE*_ is the maximum synaptic efficiency between two excitatory neurons if they are connected, and the product *J*_*EE*_*u*(*t*)*x*(*t*) denotes the instant synaptic efficacy at time *t*. *J*_*IE*_ is the synaptic efficiency from an inhibitory neuron to an excitatory one if there is a connection. We use a binary variable *w*_*ji*_ to denote the connectivity between two neurons, with *w*_*ji*_ = 1 indicating a connection from neuron *j* to *i* and *w*_*ji*_ = 0 otherwise.

For an inhibitory neuron, it receives recurrent inputs from other excitatory neurons, which is written as,

(8) $$\begin{array}{r} \tau_{s}\frac{dI_{rec,i}^{I}\left(t\right)}{dt}=-I_{rec,i}^{I}\left(t\right)+J_{EI}\underset{j\in E}{\sum }w_{ji}\underset{k}{\sum }\delta \left(t-t_{j}^{k}\right), \end{array}$$

where *J*_*EI*_ is the synapse efficiency from an excitatory neuron to an inhibitory one if there is a connection.

### 2.3. The sensory network

The sensory network is composed of *N*_*E*_ excitatory and *N*_*I*_ inhibitory neurons, with *N*_*E*_ = 4*N*_*I*_. Neurons are randomly and sparsely connected, with a probability *p* ≪ 1. All neurons receive background inputs, but only excitatory neurons receive the stimulus information directly. Only the synapses between excitatory neurons are subject to STP. Both excitatory and inhibitory neurons are connected to a downstream neuron, which reads out the stimulus information.

### 2.4. The read-out neuron

We consider that a downstream neuron read-out the stimulus information encoded in the sensory network. All neurons in the sensory network are connected to the downstream neuron. The dynamic of the downstream neuron is

(9) $$\begin{array}{r} \tau_{o}\frac{dV_{O}}{dt}=-\left(V_{O}-E_{L}\right)+R_{m}\left[J_{EO}I_{E}\left(t\right)-J_{IO}I_{I}\left(t\right)\right], \end{array}$$

where *V*_*O*_ is the membrane potential of the read-out neuron, τ_*o*_ the time constant, and *R*_*m*_ the membrane resistance. When *V*_*O*_ reaches *V*_*th*_, the read-out neuron fires. *I*_*E*_(*t*) and *I*_*I*_(*t*) are the summations of spikes from the excitatory and inhibitory groups, respectively. *J*_*EO*_ and *J*_*IO*_ represents the synaptic efficiency from the excitatory and inhibitory group to the read-out neuron. We choose *J*_*EO*_ and *J*_*IO*_ properly, such that the mean of the total input from the sensory network to the read-out neuron approximates to be zero.

### 2.5. The simulation protocol

In a single trial of simulation, we run the network dynamics for a fixed amount of time *T* = 5, 000ms. The onset of the stimulus is at *t* = 0ms. From *t* = −2, 500ms to 0ms, all neurons in the sensory network receive only background inputs, and the sensory network evolves into a stochastic stationary state, such that the response of the network to the stimulus is independent of its initial state. From *t* = 0ms to 1, 500ms, the constant stimulus is presented, and excitatory neurons in the sensory network receive a strong feed-forward input. The stimulus is terminated at *t* = 1, 500ms, and after that all sensory neurons receive only background inputs. We run the simulation for 100 trials to analyze the performance of the network. The parameters used are summarized in Table 1.

**Table 1:** Parameters used in simulations.

	**Values**
**SINGLE-NEURON PARAMETERS**	
*V*_*th, E*_ - Spiking threshold of excitatory neurons	−55 mV
*V*_*th, I*_ - Spiking threshold of inhibitory neurons	−57 mV
*E*_*L*_ - Resting potential	−65 mV
τ_*m*_ - Membrane time constant	20 ms
τ_*adp*_ - Time constant of adaptation	250 ms
*A*_*s*_ - Strength of spike-trigger adaptation	1.0 μA
*A*_*p*_ - Strength of subthreshold adaptation	0.1 (*kΩ*)^−1^
τ_*o*_ - Membrane time constant of the read-out neuron	20 ms
*R*_*m*_ - m the membrane resistance	1.0 kΩ
**SYNAPTIC PARAMETERS**	
*J*_*EE*_ - Synaptic efficacy from E to E	6 μA
*J*_*EI*_ - Synaptic efficacy from E to I	5 nA
*J*_*IE*_ - Synaptic efficacy from I to E	4 nA
τ_*s*_ - Time constant of synaptic current	5 ms
*U*- Increment of STF	0.00525
τ_*f*_ - Time constant of STF	400 ms
τ_*d*_ - Time constant of STD	1,000 ms
*J*_*s*_ - Synaptic efficacy from E to E in the static synapse model	0.129 μA
**NETWORK PARAMETERS**	
*T*- Total simulation time of a single trial	5,000 ms
*T*_*f*_ - Time window to calculate the population firing rates	100 ms
*t*_*w*_ - Time bin used to calculate firing rate distribution of the excitatory neurons	5 ms
*p*- Probability of the connection	0.1
*N*_*E*_ - Number of excitatory neurons	2,000
*N*_*I*_ - Number of inhibitory neurons	500
μ_*ext*_ -Mean of the stimulus	0.65 μA
μ_*b*_ - Mean of background input	0.45 μA
σ_*ext*_ - Standard deviation of external input	1.2 μA
*J*_*EO*_ - Synaptic efficiency from excitatory neurons to the read-out neuron	1 μA
*J*_*IO*_ - Synaptic efficiency from inhibitory neurons to the read-out neuron	2 μA

For comparison, we also shuffle synaptic currents to neurons. In a trial, for each neuron *i*, we decompose the synaptic current *c*_*i*_(*t*) to the neuron into many small bins (bin size 5 ms), and randomly shuffle the order of bins. The newly obtained current has the same mean value as *c*_*i*_(*t*), but the temporal structure of *c*_*i*_(*t*) is destroyed.

### 2.6. Measurement of correlation

In our model, STF facilitates neuronal synapses after the onset of stimulation, which increases the pair-wise correlations between neurons. The increment of neuronal correlation is rather small, nevertheless, its effect on synchronized neural population response can be significant (Bruno and Sakmann, 2006). The previous study has shown that a simple dichotomized Gaussian model can well describe how pair-wise correlations between neuronal synaptic inputs affect the synchronization of a large-size neural network (Amari et al., 2003). This model successfully predicts the high-order interactions of neural activities in the sensory cortexes (Yu et al., 2011). We therefore adopt the dichotomized Gaussian model to measure the correlation between neurons.

#### 2.6.1. The dichotomized gaussian model

Denote *s*_*i*_ to be the current received by neuron *i*, which is given by

(10) $$\begin{array}{r} s_{i}=I_{syn,i}-I_{adp,i}. \end{array}$$

Since the network size is large and neurons are randomly and sparsely connected with each other, we can approximately regard that all neurons are statistically equivalent. Moreover, in the periods of Pre-adp, Adp and Post-adp, the neural network is approximately at stationary states. According to the central limit theorem, the current *s*_*i*_ satisfies a Gaussian distribution approximately, which is written as,

(11) $$\begin{array}{r} s_{i}=\mu_{s}+\sigma_{s}\left(\sqrt{1-\alpha }v_{i}+\sqrt{\alpha }\epsilon \right), \end{array}$$

where μ_*s*_ is the mean, σ_*s*_ the standard deviation of fluctuations, and ν_*i*_ ~ *N*(0, 1), ϵ ~ *N*(0, 1) are Gaussian white noises of zero mean and unit variance, The variables ν_*i*_, for *i* = 1, …, *N*, are independent to each other, standing for input fluctuations to individual neurons.

The noise ϵ is common to all neurons, inducing correlations between neurons. It is straightforward to check that the covariance $\text{cov}\left(s_{i},s_{j}\right)=\sigma_{s}^{2}\alpha$. The previous study showed that the above simplified noise model Equation (11) well captures the high-order statistics of neural data (Amari et al., 2003; Yu et al., 2011). Therefore, we use the quantity $\sigma_{s}^{2}\alpha$ to measure the pair-wise correlation between neurons. The effect of STF is to increase $\sigma_{s}^{2}\alpha$, such that the sensory network has a higher chance to generate large-size synchronized firing.

In the dichotomized Gaussian model, neuron firing is simplified as a threshold operation, which is given by

(12) $$\begin{array}{c} x_{i}=\{\begin{array}{cc} 0,\; & s_{i}<h,\text{ silent},\\ 1,\; & s_{i}\geq h,\text{ firing}, \end{array} \end{array}$$

where *h* is the predefined threshold.

The probability density function of neural population firing rate, measured by the portion of neurons firing in an unit time is calculated to be (Amari et al., 2003)

(13) $$\begin{array}{r} p\left(r\right)=C\text{exp}\left[\frac{{\left(h-\mu_{s}-\sqrt{\alpha }\sigma_{s}F^{-1}\left(r\right)\right)}^{2}}{2\sigma_{s}^{2}\left(1-\alpha \right)}-\frac{{\left(F^{-1}\left(r\right)\right)}^{2}}{2}\right], \end{array}$$

where $F\left(\epsilon \right)=\frac{1}{\sqrt{2\pi }\sigma_{s}}\int_{\frac{h-\mu_{s}-\sigma_{s}\sqrt{\alpha }\epsilon }{\sigma_{s}\sqrt{1-\alpha }}}^{\infty }e^{\frac{-\nu^{2}}{2}}d\nu$, and *C* the normalization factor.

In the simulation, we calculated (_σ_*s*_)*i*_ for each individual neurons and take their mean as the estimate of σ_*s*_. For each neuron pair, we collected synaptic currents *s*_*i*_, *s*_*j*_ in the periods of Pre-adp, Adp and Post-adp, and calculated the covariance cov(*s*_*i*_, *s*_*j*_) in these periods, respectively. Averaging over all neuron pairs, we got the mean of pair-wise neural correlation in the network, which gives rise to $\sigma_{s}^{2}\alpha$.

## 3. Results

### 3.1. The adaptation behavior

**Figure 3:**
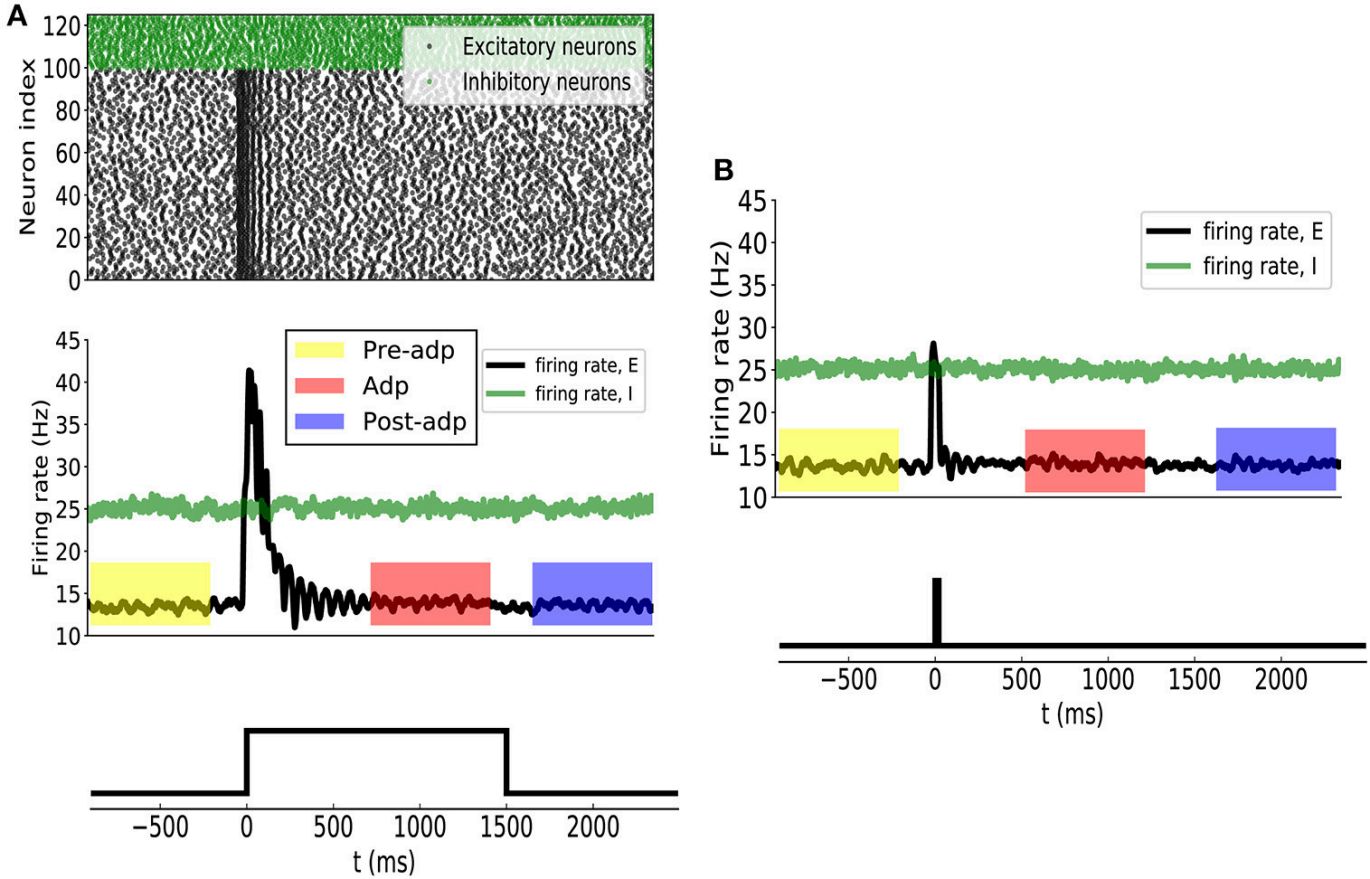
**(A)** Neural adaptation to a constant input. Stimulation is set from 0 to 1, 500 ms. (Upper panel) spiking activities of 100 example excitatory (black dots) and 25 inhibitory (green dots) example neurons. (Middle panel) the averaged firing rates of excitatory (black curve) and inhibitory (green) neuron groups. Colored boxes indicate three time periods: Pre-adp, Adp and Post-adp. Lower panel: the time course of the stimulation. **(B)** Neural responses to a transient input. Stimulation is set from 0 to 20 ms.

Before applying the stimulation, the sensory network received stochastic background inputs and was at a stationary state of low firing rates. The stimulation, which represents the presence of the stimulus via a strong external input, was applied to all excitatory neurons in the network at *t* = 0ms, and the stimulation was terminated at *t* = 1, 500ms. As shown in Figure 3A, the sensory network displayed the typical adaptive phenomenon: the firing rates of neurons first increased dramatically at the onset of the stimulation and then gradually attenuated to a level close to the background activity (after around *t* = 300ms). The length of adaptation, from the moment of firing rate increasing to the moment of firing rate returning to the background level, is mainly determined by the time constant of single neuron dynamics and the amplitude of adaptation currents. Here, we chose the parameters to let the adaptation length to be around 250ms, but generalization to other time scales is straightforward. As a comparison, when the stimulation was only presented transiently, the neuronal responses was also transient and did not exhibit the adaptation behavior (Figure 3B).

For the convenience of description, we selected three periods to analyze the response properties of the sensory network (Figure 3), which are: (1) Pre-adp: from *t* = −900ms to −100ms, 2) Adp: from *t* = 500ms to 1, 300ms, and (3) Post-adp: from *t* = 1, 600ms to 2, 400ms. In the periods of Pre-adp and Post-adp, there was no stimulation and the network activity was at the background level. In the period of Adp, although the stimulation was presented, neuronal firing rates had already attenuated to the background level. There is little difference in firing rate among three periods, but the neuronal correlations of them are different as described below.

### 3.2. The STP effect during the adaptation

The synapses between excitatory neurons in the sensory network were subject to STP. We set the time constant of STD to be large and the utilization increment of STF to be small (see section Materials and Methods), which agree with the data in the sensory cortexes (Thomson et al., 2002; Wang et al., 2006).

**Figure 4:**
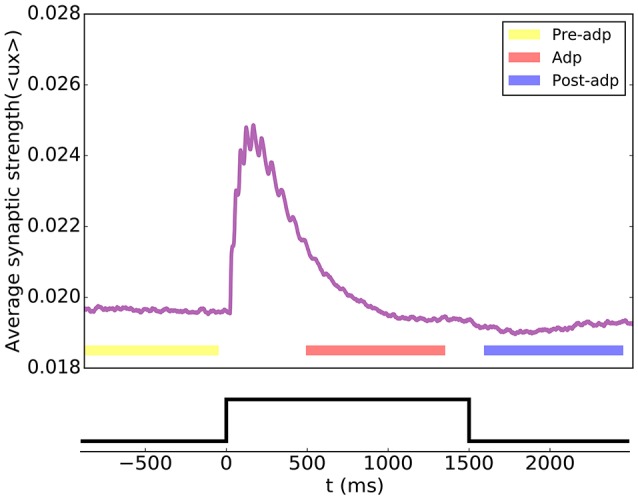
Short-term plasticity of the synapses between excitatory neurons in the sensory network. The synaptic strength of the network is measured by the average value of all synapse strengths.

According to the STP dynamics (Equation 6), the efficacy of a synapse was given by *ux*, where *u* denoted the utilization factor and *x* the fraction of available neurotransmitters. We used the mean efficacy averaged over all synapses, denoted as 〈*ux*〉, to measure the synaptic strength of the network. Figure 4 displays how the synaptic strength varied over time during the adaptation. In the period of Pre-adp, the synaptic strength of the network was at a stationary value. Immediately after the stimulation onset, the synaptic strength experiences an abrupt increase, which was due to STF triggered by strong firings of neurons. As time went on, the firing rates of neurons attenuated, and the STD effect became dominating. Since STD was a slow process, in the period of Adp, although the firing rates of neurons had attenuated, the synaptic strength of the network was still well above the background level. Finally, in the period of Post-adp, the synaptic strength of the network returned to its value before the adaptation.

### 3.3. Enhanced neural correlation during the adaptation

During the adaptation, the enhanced synaptic efficacy increased the interactions between neurons, resulting enlarged correlations between neuronal responses. This increment in neural correlation was rather small, however, its effect on the synchronized firing of the neural population (i.e., a large population of neurons firing together in a short time window) can be significant (Bruno and Sakmann, 2006). It is difficult to theoretically analyze the dynamics of the network with varying synapse strengths. Here, we adopt a simplified dichotomized Gaussian model to describe the STF effect on the synchrony of the network (Amari et al., 2003). This model estimated the neural population synchrony based on the correlation between neuronal synaptic inputs, and the latter was affected by the synaptical interactions between neurons (see section Materials and Methods). This model has been shown to fit well the characteristics of neural responses in the real neural system (Yu et al., 2011).

**Figure 5:**
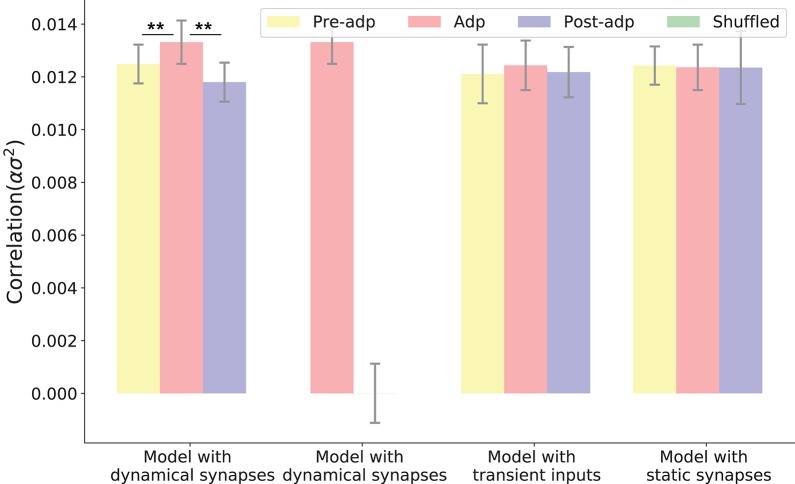
Neural correlations in different periods in different models. The neural correlation is measured by the covariance of synaptic inputs to a neuron pair averaged over the population (see section Materials and Methods). The shuffled case refers to that the synaptic currents to neurons are randomly shuffled, which destroys the temporal structures of synaptic currents but keeps their means unchanged. Only in the model with dynamic synapses, neural correlation in the period Adp is significantly larger than those in other periods. ^**^Indicates the significant difference between neural correlations, *p* < 0.05.

We simulated the sensory network for 100 trials, and measured the averaged pair-wise correlation between neurons in the network (given by $\sigma_{s}^{2}\alpha$, see Equation 11 in section Materials and Methods). Figure 5 shows the neural correlations in different periods. We saw that the neural correlation in the Adp period is larger than those in other periods (the difference is small but statistically significant), consistent with the synaptical strength differences in three periods.

To confirm that the enhanced neural correlation was really associated with STP, we carried out following comparisons. Firstly, to exclude the possibility that the increase of neural correlation was due to the variations of neuronal firing rates (which change the means of synaptic currents to neurons), we shuffled the synaptic currents to all neurons, which destroyed the temporal structures of the synaptic currents but kept their means unchanged (section Materials and Methods). We observed that in such a case, the increase of neural correlation in Adp vanished (Figure 5). Secondly, we constructed a network of static synapses. This model had the same parameters as the original one except no STP, and the synapse strength was set to be a constant, so that the neural correlations in the Pre-adp and Post-adp periods agree with those in the original model. As shown in Figure 5, without STP, the neural correlation in Adp had no significant difference with those in the other periods. Finally, we also calculated neural correlations in the case when the stimulation was only presented transiently (i.e., no adaptation, see Figure 3), and found no increase in neural correlation in Adp compared to other periods (Figure 5).

**Figure 6:**
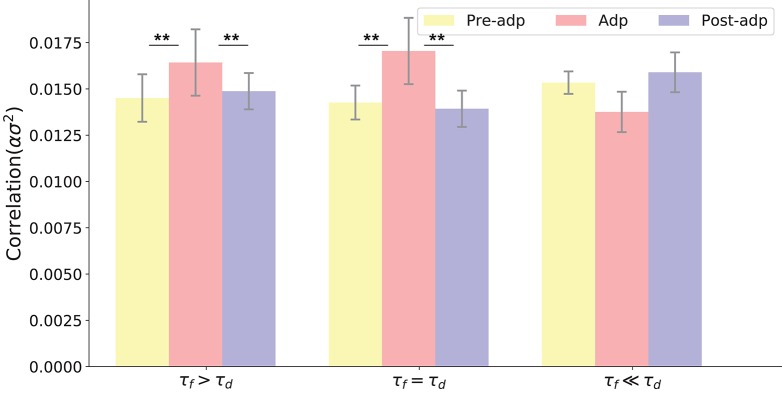
Neural correlations in different periods with varied STP parameters. Parameters used in three models (from left to right) are: τ_*d*_ = 400*ms*, τ_*f*_ = 1, 000*ms, U* = 0.0018; τ_*d*_ = 400*ms*, τ_*f*_ = 400*ms, U* = 0.00425; τ_*f*_ = 400*ms*, τ_*f*_ = 50*ms, U* = 0.02. ^**^Indicates the significant difference between neural correlations, *p* < 0.05.

We also checked the model performances for different STP parameters by choosing different combinations of τ_*f*_ and τ_*d*_, which are: τ_*f*_ = 400*ms*, τ_*d*_ = 400*ms*; τ_*f*_ = 1, 000*ms*, τ_*d*_ = 400*ms*; and τ_*f*_ = 50*ms*, τ_*d*_ = 400ms. The parameter *U* was also adjusted accordingly to ensure neuronal firing rates attenuate in the Adp period, and other parameters remained the same. Overall, we observe that the neural correlation was enhanced in the Adp period for a wide range of τ_*f*_ and only failed to increase when τ_*f*_ is too small (Figure 6).

**Figure 7:**
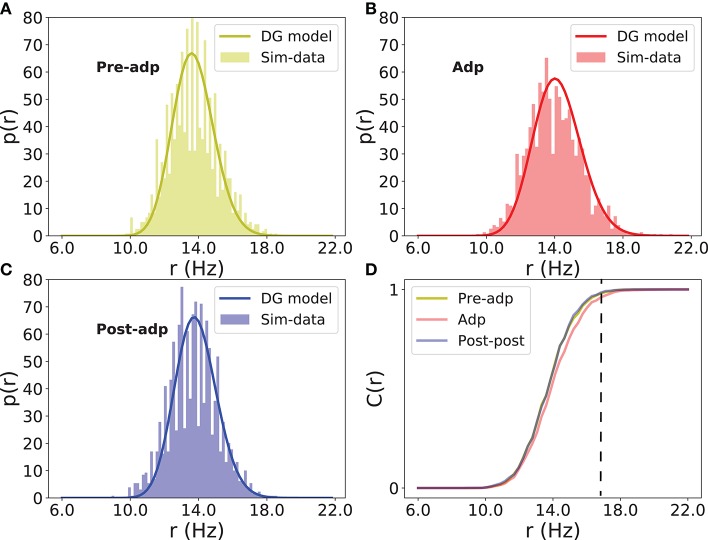
Distributions of firing rate of excitatory neurons in different periods. **(A)** Histogram of population firing rate in Pre-adp period fitted by the dichotomized Gaussian model. **(B)** Histogram of population firing rate in Adp period fitted by the dichotomized Gaussian model. **(C)** Histogram of population firing rate in Post-adp period fitted by the dichotomized Gaussian model. **(D)** Comparing population firing rates in three periods. C(r) is the cumulative distribution. Dashed line indicates that the network in Adp has a higher probability to generate large-size synchronized firing than in other two periods.

The neural correlation strength determines the probability of synchronized firing of the network. We measured the distributions of neural population firing rate in three periods by counting the portion of excitatory neurons firing in a short time bin (the bin size 5ms), and fitted these distributions by the dichotomized Gaussian model (see section Materials and Methods). We see that indeed the enhanced neural correlation increased the probability of the network generating large-size synchronized firing (Figure 7).

### 3.4. Reading-out the stimulus information

In dynamical encoding, a constant stimulation triggers adaptive responses of the stimulus-specific neurons, and during this process, although the firing rates of neurons attenuate, the correlations between neurons are enhanced, which increased the chance of the network to generate large-size synchronized firing. Thus, the synchrony of the network during the adaptation was associated with the stimulus information. Here, we show how the neural system reads out the stimulus information.

We considered a downstream neuron receives inputs from all neurons in sensory network and encodes the stimulus information (section Materials and Methods). The dynamic of the read-out neuron was given in Equation (9), and we chose the connection weights *J*_*EO*_, the synaptic efficacy from the excitatory neuron group to the downstream neuron, and *J*_*IO*_, the synaptic efficacy from the inhibitory neuron group to the downstream neuron, properly, such that the downstream neuron receives balanced synaptic inputs. The balanced condition means that the mean of the excitatory and inhibitory inputs is approximately zero, a condition which had been observed in the experiment (Shu et al., 2003). It has been suggested that the balanced condition plays important roles in neural information processing, such as to generate irregular neural spikes (Van Vreeswijk and Sompolinsky, 1996), to detect synchrony of neuronal responses (Shadlen and Newsome, 1998), and to track the change of external inputs rapidly (Van Vreeswijk and Sompolinsky, 1996). These properties come from that in the balanced condition, the membrane potential of a neuron was always close to the firing threshold, so that the neuron was sensitive to fluctuations of the input. In our model, this implies that the downstream neuron is only sensitive to the large input fluctuations triggered by synchronized firings of the sensory network, and the latter is associated with the presence of the stimulus.

**Figure 8:**
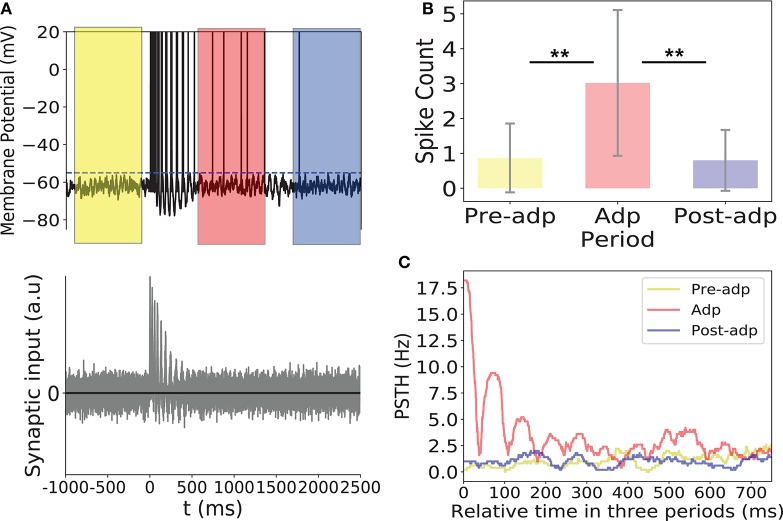
Activity of the read-out neuron. **(A)** Upper panel: membrane potential of the read-out neuron in an example trial. Dashed line is the firing threshold of the read-out neuron. Lower panel: the balanced synaptic input to the read-out neuron in an example trial. **(B)** The spike counts of the read-out neuron in different period averaged over 100 trials. **(C)** PSTH of the read-out neuron in three periods. ^**^Indicates the significant difference between spike counts, *p* < 0.05.

As shown in Figure 8, synchrony of the sensory network induced large fluctuations in the input to the read-out neuron, triggering the read-out neuron to fire. In Adp, since the chance of the sensory network to have large-size synchronized firing was much higher than in other periods, the firing rates of the read-out neuron were much larger than that in other periods (Figure 8). This ensures that the fire rate of the read-out neuron encodes the information about the presence of the stimulus reliably.

Please note that in dynamical coding, the neural system does not need separate schemes to read-out the stimulus information. For the rate code, pre-synaptic spikes arrive independently but at high frequency, so that the accumulated post-synaptic current can activate the read-out neuron. For the correlation code, pre-synaptic spikes arrive at low frequency but in a coherent manner, and the summed post-synaptic current can equally activate the read-out neuron. Thus, both codes convey the stimulus information to the same read-out neuron but in different manners as illustrated in Figure 1.

## 4. Conclusions and discussions

This study is motivated by the observation that in response to a sustained invariant stimulation, although the firing rates of individual neurons are attenuated, the neural system can still sense the presence of the stimulus. We show that this can be achieved through a dynamical encoding strategy using a computational model. At first, the stimulus information is encoded by the strong and independent firings of neurons at the early stage of adaptation; while as the firing rates attenuated due to adaptation, the information is shifted into the weak but synchronized firings of neurons. We demonstrate that STP can accomplish this shift of information encoder. In detail, the strong firings at the early stage of adaptation can facilitate the synapses between neurons via STF; thus the interactions between neurons are enhanced, causing the increase of neural correlations. This increase of neural correlations is stimulus-specific and lasts in the slow time constant of STD, and hence it can be seen as the substrate to encode the stimulus information during the adaptation, regardless of the attenuation of firing rates. Facilitated synapses can retain stimulus information, this idea has been proposed previously, for examples, it was proposed that STF contributes to hold working memory without recruiting neuronal firing in the prefrontal cortex (Mongillo et al., 2008), STF contributes to the priming effect (Gotts et al., 2012), and STP contributes to detect weak stimuli (Mejias and Torres, 2011). Here, we propose that STF contributes to implement a dynamical coding strategy in neural adaptation. Furthermore, we investigate how correlation-based information can be read out by a downstream neuron via large-size synchronized firing, and showed that the balanced inputs are crucial to implement this task efficiently.

The previous study found that adaptation of single neuron dynamics alone (without including STP of synapses) can lead to that the variability of neuronal responses in a balanced network decreases during the adaptation, which partly compensates the influence of firing rate attenuation on the reliability of neural encoding (Farkhooi et al., 2013). Here, our study goes one step further by considering the effect of STP, which increases synaptical strengthes due to the strong transient responses of neurons at the onset of stimulation, and subsequently enhances neuronal correlations during the adaptation, leading to the implementation of the dynamic encoding strategy. In such a coding scheme, the adaptation of single neuron dynamics also is not sufficient, as it does not increase neuronal correlations (as confirmed by the simulation experiment on static synapses, see Figure 5).

There has been a long standing debate on the role of correlation in neural coding (Averbeck and Lee, 2004). A few studies indicate that neural correlation conveys little stimulus information (Ecker et al., 2010; Oizumi et al., 2010; Meytlis et al., 2012); whereas, the others argue that neural correlation is crucial in the stimulus information processing. (Ishikane et al., 2005; Bruno and Sakmann, 2006; Ince et al., 2010). In this study, we reconcile these two different views. We propose a dynamical encoding strategy and we think that both views capture the characteristics of neural information encoding at the different time stages. An advantage of the correlation code is that it is economically efficient (using less spikes). One may concern that why the neural system does not employ the correlation code in the first place. An argument is that the correlation code is slow: since neurons fire weakly, it takes long time for a downstream neuron to read-out the stimulus information, but in reality, animals need to respond quickly to a newly appeared stimulus. Thus, it is likely that the brain has exploited a compensational solution: the neural system detects the appearance of a novel stimulus by using the fast firing-rate code and retains the information of a sustained stimulus by using the slow but economically more efficient correlation code. The previous study based on retina data demonstrated that enhanced electrical synapses between ganglion cells contribute to encode stimulations of different luminance levels during the adaptation (Xiao et al., 2013), but to validate the similar computational role of chemical synapses, it will be much more challenging. This is because the effect of facilitated chemical synapses on varying neuronal correlation is rather small. We hope that along with the development of neuroimaging technique, it will become eventually feasible to measure neuronal correlations over a large population of neurons *in vivo* and validate our theoretical hypothesis on dynamical neural encoding.

## Author contributions

SW and D-HW designed the project. SW and LL wrote the paper. LL, D-HW, and SW carried out simulations and data analysis. YM and WZ contributed important ideas.

### Conflict of interest statement

The authors declare that the research was conducted in the absence of any commercial or financial relationships that could be construed as a potential conflict of interest.

## References

[B1] Abbott L. F. Varela J. Sen K. Nelson S. (1997). Synaptic depression and cortical gain control. Science 275, 221–224. 10.1126/science.275.5297.221 8985017

[B2] Amari S. I. Nakahara H. Wu S. Sakai Y. (2003). Synchronous firing and higher-order interactions in neuron pool. Neural Comput. 15, 127–142. 10.1162/089976603321043720 12590822

[B3] Averbeck B. B. Lee D. (2004). Coding and transmission of information by neural ensembles. Trends Neurosci. 27, 225–230. 10.1016/j.tins.2004.02.006 15046882

[B4] Benda J. Herz A. V. (2003). A universal model for spike-frequency adaptation. Neural Comput. 15, 2523–2564. 10.1162/089976603322385063 14577853

[B5] Brette R. Gerstner W. (2005). Adaptive exponential integrate-and-fire model as an effective description of neuronal activity. J. Neurophysiol. 94, 3637–3642. 10.1152/jn.00686.2005 16014787

[B6] Bruno R. M. Sakmann B. (2006). Cortex is driven by weak but synchronously active thalamocortical synapses. Science 312, 1622–1627. 10.1126/science.1124593 16778049

[B7] Chung S. Li X. Nelson S. B. (2002). Short-term depression at thalamocortical synapses contributes to rapid adaptation of cortical sensory responses *in vivo*. Neuron 34, 437–446. 10.1016/S0896-6273(02)00659-1 11988174

[B8] Connors B. W. Long M. A. (2004). Electrical synapses in the mammalian brain. Annu. Rev. Neurosci. 27, 393–418. 10.1146/annurev.neuro.26.041002.131128 15217338

[B9] deCharms R. C. Merzenich M. M. (1996). Primary cortical representation of sounds by the coordination of action-potential timing. Nature 381:610. 10.1038/381610a0 8637597

[B10] Ecker A. S. Berens P. Keliris G. A. Bethge M. Logothetis N. K. Tolias A. S. (2010). Decorrelated neuronal firing in cortical microcircuits. Science 327, 584–587. 10.1126/science.1179867 20110506

[B11] Farkhooi F. Froese A. Muller E. Menzel R. Nawrot M. P. (2013). Cellular adaptation facilitates sparse and reliable coding in sensory pathways. PLoS Comput. Biol. 9:e1003251. 10.1371/journal.pcbi.1003251 24098101 PMC3789775

[B12] Gotts S. J. Chow C. C. Martin A. (2012). Repetition priming and repetition suppression: a case for enhanced efficiency through neural synchronization. Cogn. Neurosci. 3, 227–237. 10.1080/17588928.2012.670617 23144664 PMC3491809

[B13] Gutnisky D. A. Dragoi V. (2008). Adaptive coding of visual information in neural populations. Nature 452, 220–224. 10.1038/nature06563 18337822

[B14] Ince R. A. Senatore R. Arabzadeh E. Montani F. Diamond M. E. Panzeri S. (2010). Information-theoretic methods for studying population codes. Neural Netw. 23, 713–727. 10.1016/j.neunet.2010.05.008 20542408

[B15] Ishikane H. Gangi M. Honda S. Tachibana M. (2005). Synchronized retinal oscillations encode essential information for escape behavior in frogs. Nat. Neurosci. 8, 1087–1095. 10.1038/nn1497 15995702

[B16] Kohn A. (2007). Visual adaptation: physiology, mechanisms, and functional benefits. J. Neurophysiol. 97, 3155–3164. 10.1152/jn.00086.2007 17344377

[B17] Markram H. Wang Y. Tsodyks M. (1998). Differential signaling via the same axon of neocortical pyramidal neurons. Proc. Natl. Acad. Sci. U.S.A. 95, 5323–5328. 10.1073/pnas.95.9.5323 9560274 PMC20259

[B18] Mejias J. F. Torres J. J. (2011). Emergence of resonances in neural systems: the interplay between adaptive threshold and short-term synaptic plasticity. PLoS ONE 6:e17255. 10.1371/journal.pone.0017255 21408148 PMC3050837

[B19] Meytlis M. Nichols Z. Nirenberg S. (2012). Determining the role of correlated firing in large populations of neurons using white noise and natural scene stimuli. Vis. Res. 70, 44–53. 10.1016/j.visres.2012.07.007 22885035 PMC3980944

[B20] Middleton J. W. Omar C. Doiron B. Simons D. J. (2012). Neural correlation is stimulus modulated by feedforward inhibitory circuitry. J. Neurosci. 32, 506–518. 10.1523/JNEUROSCI.3474-11.2012 22238086 PMC3282531

[B21] Mongillo G. Barak O. Tsodyks M. (2008). Synaptic theory of working memory. Science 319, 1543–1546. 10.1126/science.1150769 18339943

[B22] Oizumi M. Ishii T. Ishibashi K. Hosoya T. Okada M. (2010). Mismatched decoding in the brain. J. Neurosci. 30, 4815–4826. 10.1523/JNEUROSCI.4360-09.2010 20357132 PMC6632316

[B23] Schneidman E. Berry M. J. II. Segev R. Bialek W. (2006). Weak pairwise correlations imply strongly correlated network states in a neural population. Nature 440:1007. 10.1038/nature04701 16625187 PMC1785327

[B24] Shadlen M. N. Newsome W. T. (1998). The variable discharge of cortical neurons: implications for connectivity, computation, and information coding. J. Neurosci. 18, 3870–3896. 9570816 10.1523/JNEUROSCI.18-10-03870.1998 PMC6793166

[B25] Shu Y. Hasenstaub A. McCormick D. A. (2003). Turning on and off recurrent balanced cortical activity. Nature 423, 288–293. 10.1038/nature01616 12748642

[B26] Thomson A. M. West D. C. Wang Y. Bannister A. P. (2002). Synaptic connections and small circuits involving excitatory and inhibitory neurons in layers 2–5 of adult rat and cat neocortex: triple intracellular recordings and biocytin labelling *in vitro*. Cereb. Cortex 12, 936–953. 10.1093/cercor/12.9.936 12183393

[B27] Van Vreeswijk C. Sompolinsky H. (1996). Chaos in neuronal networks with balanced excitatory and inhibitory activity. Science 274, 1724–1726. 10.1126/science.274.5293.1724 8939866

[B28] Wang Y. Markram H. Goodman P. H. Berger T. K. Ma J. Goldman-Rakic P. S. (2006). Heterogeneity in the pyramidal network of the medial prefrontal cortex. Nat. Neurosci. 9, 534–542. 10.1038/nn1670 16547512

[B29] Wark B. Lundstrom B. N. Fairhall A. (2007). Sensory adaptation. Curr. Opin. Neurobiol. 17, 423–429. 10.1016/j.conb.2007.07.001 17714934 PMC2084204

[B30] Xiao L. Zhang M. Xing D. Liang P. J. Wu S. (2013). Shifted encoding strategy in retinal luminance adaptation: from firing rate to neural correlation. J. Neurophysiol. 110, 1793–1803. 10.1152/jn.00221.2013 23864383

[B31] Xiao L. Zhang P. M. Wu S. Liang P. J. (2014). Response dynamics of bullfrog on-off rgcs to different stimulus durations. J. Comput. Neurosci. 37, 149–160. 10.1007/s10827-013-0492-2 24390227

[B32] Yu S. Yang H. Nakahara H. Santos G. S. Nikolić D. Plenz D. (2011). Higher-order interactions characterized in cortical activity. J. Neurosci. 31, 17514–17526. 10.1523/JNEUROSCI.3127-11.2011 22131413 PMC6623824

